# An existence-uniqueness theorem and alternating contraction projection methods for inverse variational inequalities

**DOI:** 10.1186/s13660-018-1943-0

**Published:** 2018-12-18

**Authors:** Songnian He, Qiao-Li Dong

**Affiliations:** College of Science, Civil Aviation University of China, Tianjin, China

**Keywords:** 47J20, 90C25, 90C30, 90C52, Inverse variational inequality, Variational inequality, Lipschitz continuous, Strongly monotone

## Abstract

Let *C* be a nonempty closed convex subset of a real Hilbert space $\mathcal{H}$ with inner product $\langle \cdot , \cdot \rangle $, and let $f: \mathcal{H}\rightarrow \mathcal{H}$ be a nonlinear operator. Consider the inverse variational inequality (in short, $\operatorname{IVI}(C,f)$) problem of finding a point $\xi ^{*}\in \mathcal{H}$ such that

$$ f\bigl(\xi ^{*}\bigr)\in C, \quad \bigl\langle \xi ^{*}, v-f \bigl(\xi ^{*}\bigr)\bigr\rangle \geq 0, \quad \forall v\in C. $$

In this paper, we prove that $\operatorname{IVI}(C,f)$ has a unique solution if *f* is Lipschitz continuous and strongly monotone, which essentially improves the relevant result in (Luo and Yang in Optim. Lett. 8:1261–1272, 2014). Based on this result, an iterative algorithm, named the alternating contraction projection method (ACPM), is proposed for solving Lipschitz continuous and strongly monotone inverse variational inequalities. The strong convergence of the ACPM is proved and the convergence rate estimate is obtained. Furthermore, for the case that the structure of *C* is very complex and the projection operator $P_{C}$ is difficult to calculate, we introduce the alternating contraction relaxation projection method (ACRPM) and prove its strong convergence. Some numerical experiments are provided to show the practicability and effectiveness of our algorithms. Our results in this paper extend and improve the related existing results.

## Introduction

Let $\mathcal{H}$ be a real Hilbert space with inner product $\langle \cdot ,\cdot \rangle $ and induced norm $\Vert \cdot \Vert $. Recall that the metric projection operator of a nonempty closed convex subset *C* of $\mathcal{H}$, $P_{C}:\mathcal{H}\rightarrow C$, is defined by

$$ P_{C}(x):=\arg \min_{y\in C} \Vert x-y \Vert ^{2}, \quad x\in \mathcal{H}. $$

Let *C* be a nonempty closed convex subset of $\mathcal{H}$, and let $F: C\rightarrow \mathcal{H}$ be a nonlinear operator. The so-called variational inequality (in short, $\operatorname{VI}(C,F)$) problem is to find a point $u^{*}\in C$ such that

1 $$ \bigl\langle F\bigl(u^{*}\bigr),v-u^{*}\bigr\rangle \geq 0, \quad \forall v\in C. $$

The variational inequalities have many important applications in different fields and have been studied intensively, see [1, 2, 4, 7–14, 17, 24, 26, 27, 31, 32, 36, 38–40, 42–46], and the references therein.

It is easy to verify that $u^{*}$ solves $\operatorname{VI}(C,F)$ if and only if $u^{*}$ is a solution of the fixed point equation

2 $$ u^{*}=P_{C}(I-\lambda F)u^{*}, $$

where *I* is the identity operator on $\mathcal{H}$ and *λ* is an arbitrary positive constant.

A class of variant variational inequalities is the inverse variational inequality (in short $\operatorname{IVI}(C,f)$) problem [19], which is to find a point $\xi ^{*}\in \mathcal{H}$ such that

3 $$ f\bigl(\xi ^{*}\bigr)\in C, \quad \bigl\langle \xi ^{*}, v-f\bigl(\xi ^{*}\bigr)\bigr\rangle \geq 0, \quad \forall v\in C, $$

where $f: \mathcal{H}\rightarrow \mathcal{H}$ is a nonlinear operator. The inverse variational inequalities are also widely used in many different fields such as the transportation system operation, control policies, and the electrical power network management [20, 22, 41].

Now we give a brief overview of the properties and algorithms of inverse variational inequalities. For the properties of inverse variational inequalities, Han et al. [16] proved that the solution set of any monotone inverse variational inequality is convex. He [18] proved that the inverse variational inequality $\operatorname{IVI}(C,f)$ is equivalent to the following projection equation:

$$ f\bigl(\xi ^{*}\bigr)=P_{C}\bigl(f\bigl(\xi ^{*} \bigr)-\beta \xi ^{*}\bigr), $$

where *β* is an arbitrary positive constant. Consequently, the problem $\operatorname{IVI}(C,f)$ equals the fixed point problem of the mapping

$$ T:=I-f+P_{C}(f-\beta I). $$

The following lemma reveals the intrinsic relationship between variational inequalities and inverse variational inequalities.

### Lemma 1.1

([23, 34])

*If* $f: \mathcal{H}\rightarrow \mathcal{H}$ *is a one*-*to*-*one correspondence*, *then* $\xi ^{*}\in \mathcal{H}$ *is a solution of* $\operatorname{IVI}(C,f)$ *if and only if* $u^{*}=f(\xi ^{*})$ *is a solution of* $\operatorname{VI}(C,f ^{-1})$.

As for the existence and uniqueness of solutions for Lipschitz continuous and strongly monotone inverse variational inequalities, Luo et al. [34] proved the following result.

### Lemma 1.2

([34, Lemma 1.3])

*If* $f: \mathcal{H}\rightarrow \mathcal{H}$ *is* *L*-*Lipschitz continuous and* *η*-*strongly monotone*, *and there exists some positive constant* *β* *such that*

4 $$\begin{aligned}& \vert \beta -\eta \vert < \sqrt{\eta ^{2}-2\eta +2-2 \sqrt{1-2\eta +L^{2}}}, \end{aligned}$$

5 $$\begin{aligned}& \eta ^{2}-2\eta +2-2\sqrt{1-2\eta +L^{2}}>0, \end{aligned}$$

*and*

6 $$ \eta \leq L< \sqrt{2\eta }, $$

*then* *T* *is a strict contraction with the coefficient*

$$ \sqrt{1-2\eta +L^{2}}+\sqrt{L^{2}-2\beta \eta +\beta ^{2}}< 1. $$

*Hence the inverse variational inequality* $\operatorname{IVI}(C,f)$ *has one and only one solution*.

It is easy to see that conditions (4)–(6) are not only rather harsh, but also nonessential.

The main iterative algorithms to approximate the inverse variational inequalities (3) are projection methods [28]. He et al. [21, 23] introduced PPA-based methods, exact proximal point algorithm and inexact proximal point algorithm, for monotone inverse variational inequalities and constrained ‘black-box’ inverse variational inequalities, respectively. They also gave the prediction-correction proximal point algorithm and the adaptive prediction-correction proximal point algorithm. Under certain conditions, the convergence rate of these algorithms is proved to be linear. Based on Lemma 1.2, Luo et al. [34] introduced several regularized iterative algorithms to solve monotone and Lipschitz continuous inverse variational inequalities.

There is also a lot of research on the properties of the inverse variational inequalities. We refer the reader to the papers [29, 30, 35, 37], and the references therein for the well-posedness of inverse variational inequalities. Very recently, Chen et al. [6] obtained the optimality conditions for solutions of constrained inverse vector variational inequalities by means of nonlinear scalarization.

Although inverse variational inequalities have a wide range of applications, they have not received enough attention. For example, some fundamental problems, including the existence and uniqueness of solutions, still need further study.

In this paper, based on Lemma 1.1, we firstly prove that $\operatorname{IVI}(C,f)$ has a unique solution if *f* is Lipschitz continuous and strongly monotone. This means that conditions (4)–(6) are all redundant and therefore can be eliminated. By making full use of the existing results, an iterative algorithm, named alternating contraction projection method (ACPM), is proposed for solving Lipschitz continuous and strongly monotone inverse variational inequalities. The strong convergence of the ACPM is proved and the convergence rate estimate is obtained. Furthermore, for the case that the structure of *C* is very complex and the projection operator $P_{C}$ is difficult to calculate, we introduce the alternating contraction relaxation projection method (ACRPM) and prove its strong convergence. Some numerical experiments, which show advantages of the proposed algorithms, are provided. The results in this paper extend and improve the related existing results.

## Preliminaries

In this section, we list some concepts and tools that will be used in the proofs of the main results. In the sequel, we use the notations:

(i) → denotes strong convergence;
(ii) ⇀ denotes weak convergence;
(iii) $\omega _{w}(x_{n}) =\{x\mid \exists \{x_{n_{k}}\}_{k=1} ^{\infty }\subset \{x_{n}\}_{n=1}^{\infty }\text{ such that } x_{n_{k}} \rightharpoonup x\}$ denotes the weak *ω-limit* set of $\{x_{n}\}_{n=1}^{\infty }$.

The next inequality is trivial but in common use.

### Lemma 2.1

$$ \Vert x+y \Vert ^{2}\leq \Vert x \Vert ^{2}+2\langle y,x+y \rangle , \quad \forall x,y\in \mathcal{H}. $$

### Definition 2.1

Let $f: \mathcal{H}\rightarrow \mathcal{H}$ be a single-valued mapping. *f* is said to be

(i) monotone if
  $$ \bigl\langle f(x)-f(y),x-y \bigr\rangle \geq 0, \quad \forall x, y\in \mathcal{H}; $$
(ii) *η*-strongly monotone if there exists a constant $\eta > 0$ such that
  $$ \bigl\langle f(x)-f(y),x-y \bigr\rangle \geq \eta \Vert x-y \Vert ^{2} , \quad \forall x, y \in \mathcal{H}; $$
(iii) *L*-Lipschitz continuous if there exists a constant $L > 0$ such that
  $$ \bigl\Vert f(x)-f(y) \bigr\Vert \leq L \Vert x-y \Vert , \quad \forall x, y\in \mathcal{H}; $$
(iv) nonexpansive if
  $$ \bigl\Vert f(x)-f(y) \bigr\Vert \leq \Vert x-y \Vert , \quad \forall x, y\in \mathcal{H}; $$
(v) firmly nonexpansive if
  $$ \bigl\Vert f(x)-f(y) \bigr\Vert ^{2}\leq \Vert x-y \Vert ^{2}- \bigl\Vert \bigl(x-f(x)\bigr)-\bigl(y-f(y)\bigr) \bigr\Vert ^{2} , \quad \forall x, y\in \mathcal{H}. $$

It is well known that $P_{C}$ is also firmly nonexpansive.

For the projection operator $P_{C}$, the following characteristic inequality holds.

### Lemma 2.2

([15, Sect. 3])

*Let* $z\in \mathcal{H}$ *and* $u\in C$. *Then* $u=P_{C}z$ *if and only if*

$$ \langle z-u,v-u\rangle \leq 0, \quad \forall v\in C . $$

By using Lemma 2.2 and the definition of variational inequality (1), we get the following results.

### Lemma 2.3

$u\in C$ *is a solution of* $\operatorname{VI}(C,F)$ *if and only if* *u* *satisfies the fixed*-*point equation*

$$ u=P_{C}(I-\mu F)u, $$

*where* *μ* *is an arbitrary positive constant*.

### Lemma 2.4

([5, Theorem 5])

*Let* *C* *be a nonempty closed convex subset of a real Hilbert space* $\mathcal{H}$, *and let* $F: \mathcal{H}\rightarrow \mathcal{H}$ *be* *L*-*Lipschitz continuous and* *η*-*strongly monotone*. *Let* *λ* *and* *μ* *be constants such that* $\lambda \in (0,1) $ *and* $\mu \in (0, \frac{2\eta }{L^{2}})$, *respectively*, *and let* $T^{\mu }=P_{C}( I-\mu F)$ (*or* $I-\mu F $) *and* $T^{\lambda ,\mu }=P _{C}( I-\lambda \mu F)$ (*or* $I-\lambda \mu F $). *Then* $T^{\mu }$ *and* $T^{\lambda ,\mu } $ *are all strict contractions with coefficients* $1-\tau $ *and* $1-\lambda \tau $, *respectively*, *where* $\tau = \frac{1}{2}\mu (2\eta -\mu L^{2})$.

The following two lemmas are crucial for the analysis of the proposed algorithms.

### Lemma 2.5

([33])

*Assume that* $\{a_{n}\}_{n=0}^{\infty }$ *is a sequence of nonnegative real numbers such that*

$$ a_{n+1}\leq (1-\gamma _{n})a_{n}+\gamma _{n}\delta _{n}, \quad n \geq 0, $$

*where* $\{\gamma _{n}\}_{n=0}^{\infty }$ *is a sequence in* (0,1) *and* $\{\delta _{n}\}_{n=0}^{\infty }$ *is a real sequence such that*

(i) $\sum_{n=0}^{\infty } \gamma _{n}=\infty $;
(ii) $\limsup_{n\rightarrow \infty }\delta _{n} \leq 0 $ *or* $\sum_{n=0}^{\infty } \vert \gamma _{n}\delta _{n} \vert <\infty $.

*Then* $\lim_{n\rightarrow \infty } a_{n}=0$.

### Lemma 2.6

([27])

*Assume that* $\{s_{n}\}_{n=0}^{\infty }$ *is a sequence of nonnegative real numbers such that*

$$\begin{aligned}& s_{n+1}\leq (1-\gamma _{n})s_{n}+\gamma _{n}\delta _{n}, \quad n\geq 0, \\& s_{n+1}\leq s_{n}-\eta _{n}+\alpha _{n}, \quad n\geq 0, \end{aligned}$$

*where* $\{\gamma _{n}\}_{n=0}^{\infty }$ *is a sequence in* $(0,1)$, $\{\eta _{n}\}_{n=0}^{\infty }$ *is a sequence of nonnegative real numbers*, *and* $\{\delta _{n}\}_{n=0}^{\infty }$ *and* $\{\alpha _{n}\}_{n=0} ^{\infty }$ *are two sequences in* $\mathbb{R}$ *such that*

(i) $\sum_{n=0}^{\infty } \gamma _{n}=\infty $,
(ii) $\lim_{n\rightarrow \infty } \alpha _{n}=0$,
(iii) $\lim_{k\rightarrow \infty }\eta _{n_{k}}=0$ *implies* $\limsup_{k\rightarrow \infty }\delta _{n_{k}}\leq 0$ *for any subsequence* $\{n_{k}\}_{k=0}^{\infty }\subset \{n\}_{n=0}^{\infty }$.

*Then* $\lim_{n\rightarrow \infty } s_{n}=0$.

Recall that a function $\varphi : \mathcal{H}\rightarrow \mathbb{R}$ is called convex if

$$ \varphi \bigl(\lambda u+(1-\lambda )v\bigr)\leq \lambda \varphi (u)+(1-\lambda ) \varphi (v), \quad \forall \lambda \in [0,1], \forall u, v\in \mathcal{H}. $$

Recall that an element $g\in \mathcal{H}$ is said to be a subgradient of a convex function $\varphi : \mathcal{H} \rightarrow \mathbb{R}$ at *u* if

7 $$ \varphi (z)\geq \varphi (u)+\langle g,z-u\rangle , \quad \forall z\in \mathcal{H}. $$

A convex function $\varphi : \mathcal{H}\rightarrow \mathbb{R}$ is said to be subdifferentiable at *u*, if it has at least one subgradient at *u*. The set of subgradients of *φ* at *u* is called the subdifferential of *φ* at *u*, which is denoted by $\partial \varphi (u)$. Relation (7) is called the subdifferential inequality of *φ* at *u*. A function *φ* is called subdifferentiable, if it is subdifferentiable at every $u\in \mathcal{H}$. If a convex function *φ* is differentiable, then its gradient and subgradient coincide.

Recall that a function $\varphi : \mathcal{H}\rightarrow \mathbb{R}$ is said to be weakly lower semi-continuous (*w-lsc*) at *u* if $u_{n}\rightharpoonup u$ implies

$$ \varphi (u)\leq \liminf_{n\rightarrow \infty }\varphi (u_{n}). $$

## An existence and uniqueness theorem

In this section, with the help of Lemma 1.1, an existence and uniqueness theorem of solutions for inverse variational inequalities is established.

Firstly, applying Lemma 2.3, Lemma 2.4, and Banach’s contraction mapping principle, it is not difficult to get the following well-known result.

### Theorem 3.1

*Let* *C* *be a nonempty closed convex subset of a real Hilbert space* $\mathcal{H}$, *and let* $F: C \rightarrow \mathcal{H}$ *be a Lipschitz continuous and strongly monotone operator*. *Then the variational inequality* $\operatorname{VI}(C,F)$ *has a unique solution*. *Furthermore*, *if* $F: C \rightarrow \mathcal{H}$ *is* *L*-*Lipschitz continuous and* *η*-*strongly monotone*, *then for any* $\mu \in (0, \frac{2\eta }{L ^{2}})$, $P_{C}(I-\mu F): C\rightarrow C$ *is a strict contraction and the sequence* $\{x_{n}\}_{n=0}^{\infty }$ *generated by the gradient projection method*

8 $$ x_{n+1}=P_{C}(I-\mu F)x_{n} $$

*converges strongly to the unique solution of* $\operatorname{VI}(C,F)$, *where the initial guess* $x_{0}$ *can be selected in* $\mathcal{H}$ *arbitrarily*.

Secondly, we show the following two facts.

### Lemma 3.1

*If* $f: \mathcal{H}\rightarrow \mathcal{H}$ *is Lipschitz continuous and strongly monotone*, *then* $f: \mathcal{H}\rightarrow \mathcal{H}$ *is a bijection and thus* $f^{-1}: \mathcal{H}\rightarrow \mathcal{H}$ *is a single*-*valued mapping*.

### Proof

In order to complete the proof, it suffices to verify that, for any $v\in \mathcal{H}$, there exists only one $u\in \mathcal{H}$ such that $f(u)=v$. Suppose that *f* is *L*-Lipschitz continuous and *η*-strongly monotone with $L>0$ and $\eta >0$. Take $\mu \in (0, \frac{2\eta }{L^{2}})$ and set $T=(I-\mu f)+\mu v: \mathcal{H}\rightarrow \mathcal{H}$. It is easy to verify that $u\in \mathcal{H}$ solves the equation $f(u)=v$ if and only if $u\in \mathcal{H}$ is a fixed point of *T*. Using Lemma 2.4, *T* is a strict contraction and hence *T* has only one fixed point. Consequently, the equation $f(u)=v$ has only one solution and this completes the proof. □

### Lemma 3.2

*If* $f:\mathcal{H}\rightarrow \mathcal{H}$ *is* *L*-*Lipschitz continuous and* *η*-*strongly monotone*, *then* $f^{-1}: \mathcal{H}\rightarrow \mathcal{H}$ *is* $\frac{1}{\eta }$-*Lipschitz continuous and* $\frac{ \eta }{L^{2}}$-*strongly monotone*.

### Proof

For any $x,y\in \mathcal{H}$, setting $f^{-1}(x)=u$ and $f^{-1}(y)=v$, and using the strong monotonicity of *f*, we have

$$ \begin{aligned} \bigl\Vert f^{-1}(x)-f^{-1}(y) \bigr\Vert ^{2} &= \Vert u-v \Vert ^{2} \\ &\leq \frac{1}{\eta } \bigl\langle f(u)-f(v),u-v \bigr\rangle \\ & = \frac{1}{\eta }\bigl\langle x-y, f ^{-1}(x)-f^{-1}(y) \bigr\rangle \\ &\leq \frac{1}{\eta } \Vert x-y \Vert \cdot \bigl\Vert f ^{-1}(x)-f^{-1}(y) \bigr\Vert . \end{aligned} $$

Consequently,

$$ \bigl\Vert f^{-1}(x)-f^{-1}(y) \bigr\Vert \leq \frac{1}{\eta } \Vert x-y \Vert , $$

which implies that $f^{-1}$ is $\frac{1}{\eta }$-Lipschitz continuous.

On the other hand, noting that *f* is *L*-Lipschitz continuous, we obtain

$$ \begin{aligned} \bigl\langle f^{-1}(x)-f^{-1}(y),x-y \bigr\rangle &= \bigl\langle u-v, f(u)-f(v) \bigr\rangle \\ &\geq \eta \Vert u-v \Vert ^{2} \\ &\geq \frac{\eta }{L^{2}} \bigl\Vert f(u)-f(v) \bigr\Vert ^{2}= \frac{\eta }{L^{2}} \Vert x-y \Vert ^{2}, \end{aligned} $$

which yields that $f^{-1}$ is $\frac{\eta }{L^{2}}$-strongly monotone. □

### Theorem 3.2

*Let* *C* *be a nonempty closed convex subset of a real Hilbert space* $\mathcal{H}$, *and let* $f: \mathcal{H}\rightarrow \mathcal{H}$ *be a Lipschitz continuous and strongly monotone operator*. *Then the inverse variational inequality* $\operatorname{IVI}(C,f)$ *has a unique solution*.

### Proof

From Lemma 3.1 and Lemma 3.2, we have that $f^{-1}$ is single-valued, $\frac{1}{\eta }$-Lipschitz continuous, and $\frac{\eta }{L^{2}}$-strongly monotone. Thus, by using Theorem 3.1, we assert that $\operatorname{VI}(C, f^{-1})$ has a unique solution. From Lemma 1.1, it follows that the inverse variational inequality problem $\operatorname{IVI}(C,f)$ also has a unique solution. □

### Remark 3.1

In Theorem 3.2, we just need that $f: \mathcal{H}\rightarrow \mathcal{H}$ is Lipschitz continuous and strongly monotone and do not need (4)–(6). So, our result essentially improves Lemma 1.2.

## An alternating contraction projection method

Let *C* be a nonempty closed convex subset of a real Hilbert space $\mathcal{H}$, and let $f: \mathcal{H}\rightarrow \mathcal{H}$ be an *L*-Lipschitz continuous and *η*-strongly monotone operator. Using Theorem 3.2, we assert that the inverse variational inequality $\operatorname{IVI}(C,f)$ has a unique solution, which is denoted by $\xi ^{*}$. According to Lemma 1.1, $u^{*}=f(\xi ^{*})$ is the unique solution of $\operatorname{VI}(C,f^{-1})$. Based on this fundamental fact and the gradient projection method for solving $\operatorname{VI}(C,f^{-1})$, in this section, we introduce an iterative algorithm for finding the unique solution $\xi ^{*}$ of $\operatorname{IVI}(C,f)$.

Set $\tilde{L}=\frac{ 1}{ \eta }$ and $\tilde{\eta }=\frac{ \eta }{ L^{2}}$. Take two positive constants *μ* and *α* such that $0 < \mu <\frac{ 2\tilde{\eta }}{ \tilde{L^{2}}}$ and $0 < \alpha < \frac{ 2\eta }{ L^{2}}$, respectively and a sequence of positive numbers $\{ \varepsilon _{n}\}_{n=0}^{\infty }$ such that $\varepsilon _{n}\rightarrow 0$ as $n \rightarrow \infty $. The alternating contraction projection method (ACPM) is defined as follows.

### Algorithm 4.1

(The alternating contraction projection method)

1. Take $u_{0}\in C$ and $\xi _{0}^{(0)}\in \mathcal{H}$ arbitrarily and set $n:=0$.
2. For the current $u_{n}$ and $\xi _{n}^{(0)}$ ($n\geq 0$), calculate
  9 $$ \xi ^{(m+1)}_{n}=\xi ^{(m)}_{n}- \alpha f\bigl(\xi ^{(m)}_{n} \bigr)+\alpha u_{n}, \quad m=0,1,\ldots,m _{n}, $$
  where $m_{n}$ is the smallest positive integer such that
  10 $$ \frac{(1-\tau )^{m_{n}+1}}{\tau } \bigl\Vert \xi ^{(1)}_{n}- \xi ^{(0)}_{n} \bigr\Vert \leq \varepsilon _{n}, $$
  where $\tau =\frac{1}{2}\alpha (2\eta -\alpha L^{2})$.
  Set
  11 $$ \xi _{n}=\xi ^{(m_{n}+1)}_{n}. $$
3. Calculate
  12 $$ u_{n+1}=P_{C}(u_{n}-\mu \xi _{n}), $$
  and set
  13 $$ \xi ^{(0)}_{n+1}=\xi _{n}, $$
  $n:=n+1$ and return to Step 2.

We now establish the strong convergence of Algorithm 4.1.

### Theorem 4.1

*Let* *C* *be a nonempty closed convex subset of a real Hilbert space* $\mathcal{H}$, *and let* $f: \mathcal{H}\rightarrow \mathcal{H}$ *be an* *L*-*Lipschitz continuous and* *η*-*strongly monotone operator*. *Then the two sequences* $\{\xi _{n}\}_{n=0}^{\infty }$ *and* $\{u_{n}\}_{n=0} ^{\infty }$ *generated by Algorithm *4.1 *converge strongly to the unique solution* $\xi ^{*} $ *of* $\operatorname{IVI}(C,f)$ *and the unique solution* $u^{*}$ *of* $\operatorname{VI}(C,f^{-1})$, *respectively*.

### Proof

First of all, for each $n\geq 0$ and $u_{n}\in C$, we define a mapping $T_{n}: \mathcal{H}\rightarrow \mathcal{H}$ by

14 $$ T_{n}(\xi )=(I-\alpha f) (\xi )+\alpha u_{n}, \quad \forall \xi \in \mathcal{H}. $$

From Lemma 2.4, $T_{n}$ is a strict contraction with the coefficient $1-\tau $. Moreover, Banach’s contraction mapping principle implies that the sequence $\{\xi _{n}^{(m)}\}_{m=0}^{\infty }$ generated by (9) converges strongly to $f^{-1}(u_{n})$ as $m\rightarrow \infty $ and there exists the error estimate

$$ \bigl\Vert \xi _{n}^{(m)}-f^{-1}(u_{n}) \bigr\Vert \leq \frac{(1-\tau )^{m}}{\tau } \bigl\Vert \xi _{n}^{(1)}- \xi _{n}^{(0)} \bigr\Vert ,\quad m\geq 1. $$

From (9), we have

15 $$ \bigl\Vert \xi _{n}-f^{-1}(u_{n}) \bigr\Vert = \bigl\Vert \xi _{n}^{(m_{n}+1)}-f^{-1}(u_{n}) \bigr\Vert \leq \frac{(1- \tau )^{m_{n}+1}}{\tau } \bigl\Vert \xi _{n}^{(1)}- \xi _{n}^{(0)} \bigr\Vert \leq \varepsilon _{n} . $$

Secondly, using Lemma 2.4 again, we claim that the mapping $P_{C}(I-\mu f^{-1}): C\rightarrow C$ is also a strict contraction with the coefficient $1-\tilde{\tau }$, where $\tilde{\tau }=\frac{1}{2}\mu (2\tilde{\eta }-\mu \tilde{L}^{2})$. Based on these facts and noting $u^{*}=P_{C}(u^{*}-\mu f^{-1}(u^{*}))$, we have from (15) that

16 $$ \begin{aligned}[b] \bigl\Vert u_{n+1}-u^{*} \bigr\Vert &\leq \bigl\Vert P_{C}(u_{n}-\mu \xi _{n})-P_{C}\bigl(u_{n}- \mu f^{-1}(u_{n}) \bigr) \bigr\Vert \\ & \quad {} + \bigl\Vert P_{C}\bigl(u_{n}-\mu f^{-1}(u_{n}) \bigr)- P_{C}(u ^{*}-\mu f^{-1}\bigl(u^{*} \bigr) \bigr\Vert \\ &\leq \mu \bigl\Vert \xi _{n}-f^{-1}(u_{n}) \bigr\Vert +(1- \tilde{\tau }) \bigl\Vert u_{n}-u^{*} \bigr\Vert \\ &\leq (1-\tilde{\tau }) \bigl\Vert u_{n}-u^{*} \bigr\Vert +\mu \varepsilon _{n}. \end{aligned} $$

Applying Lemma 2.5 to (16), we obtain that $\Vert u_{n}-u^{*} \Vert \rightarrow 0$ as $n\rightarrow \infty $.

Finally, noting that $\xi ^{*}=f^{-1}(u^{*})$ and $f^{-1}$ is *L̃*-Lipschitz continuous, we have from (15) that

17 $$ \begin{aligned}[b] \bigl\Vert \xi _{n}- \xi ^{*} \bigr\Vert &\leq \bigl\Vert \xi _{n}- f^{-1}(u_{n}) \bigr\Vert + \bigl\Vert f^{-1}(u_{n})- \xi ^{*} \bigr\Vert \\ &\leq \varepsilon _{n}+ \bigl\Vert f^{-1}(u_{n})-f^{-1} \bigl(u^{*}\bigr) \bigr\Vert \\ & \leq \varepsilon _{n}+\tilde{L} \bigl\Vert u_{n}-u^{*} \bigr\Vert . \end{aligned} $$

Thus it concludes from (17) that $\Vert \xi _{n}-\xi ^{*} \Vert \rightarrow 0$ holds as $n\rightarrow \infty $. □

As for the convergence rate of Algorithm 4.1, we have the following result.

### Theorem 4.2

*Under the conditions of Theorem *4.1, *we obtain the following estimates of convergence rate for Algorithm *4.1:

18 $$ \bigl\Vert u_{n}-u^{*} \bigr\Vert \leq (1-\tilde{\tau })^{n} \bigl\Vert u_{0}-u^{*} \bigr\Vert +\mu \sum_{k=0} ^{n-1}(1-\tilde{ \tau })^{n-1-k}\varepsilon _{k}, \quad \forall n\geq 1, $$

*and*

19 $$ \bigl\Vert \xi _{n}-\xi ^{*} \bigr\Vert \leq \varepsilon _{n}+\tilde{L}\Biggl\{ (1-\tilde{\tau })^{n} \bigl\Vert u_{0}-u^{*} \bigr\Vert +\mu \sum _{k=0}^{n-1}(1-\tilde{\tau })^{n-1-k} \varepsilon _{k}\Biggr\} , \quad \forall n\geq 1. $$

*In particular*, *if we take* $\varepsilon _{n}=(1- \tilde{\tau })^{n+1}$ ($n\geq 0$), *then there hold*

20 $$ \bigl\Vert u_{n}-u^{*} \bigr\Vert \leq \bigl[ \bigl\Vert u_{0}-u^{*} \bigr\Vert +\mu n\bigr](1- \tilde{\tau })^{n}, \quad \forall n\geq 1, $$

*and*

21 $$ \bigl\Vert \xi _{n}-\xi ^{*} \bigr\Vert \leq \bigl\{ (1-\tilde{\tau })+\tilde{L}\bigl[ \bigl\Vert u_{0}-u^{*} \bigr\Vert + \mu n\bigr]\bigr\} (1-\tilde{\tau })^{n}, \quad \forall n\geq 1. $$

### Proof

Estimate (18) can be obtained easily by using (16) repeatedly. By combining (18) and (17), we have (19). (20) and (21) can be gotten by substituting $\varepsilon _{n}=(1- \tilde{\tau })^{n+1}$ into (18) and (19), respectively. □

## An alternating contraction relaxation projection method

Algorithm 4.1 (ACPM) can be well implemented if the structure of the set *C* is very simple and the projection operator $P_{C}$ is easy to calculate. However, the calculation of a projection onto a closed convex subset is generally difficult. To overcome this difficulty, Fukushima [13] suggested a relaxation projection method to calculate the projection onto a level set of a convex function by computing a sequence of projections onto half-spaces containing the original level set. Since its inception, the relaxation technique has received much attention and has been used by lots of authors to construct iterative algorithms for solving nonlinear problems, see [25] and the references therein.

We now consider the inverse variational inequality problem $\operatorname{IVI}(C,f)$, where $f: \mathcal{H}\rightarrow \mathcal{H}$ is a Lipschitz continuous and strongly monotone operator. Let the closed convex subset *C* be the level set of a convex function, i.e.,

22 $$ C=\bigl\{ z\in \mathcal{H}\mid c(x)\leq 0\bigr\} , $$

where $c: \mathcal{H}\rightarrow \mathbb{R}$ is a convex function. We always assume that *c* is weakly lower semi-continuous, subdifferentiable on $\mathcal{H}$, and *∂c* is a bounded operator (i.e., bounded on bounded sets). It is worth noting that the subdifferential operator is bounded for a convex function defined on a finite dimensional Hilbert space (see [3, Corollary 7.9]).

Take the constants $\tilde{L}=\frac{1}{\eta }$, $\tilde{\eta }=\frac{\eta }{L^{2}}$, *μ*, and *α* and the sequence of positive numbers $\{\varepsilon _{n}\}_{n=0}^{\infty }$ as in the last section.

Adopting the relaxation technique of Fukushima [13], we introduce a relaxed projection algorithm for computing the unique solution $\xi ^{*}$ of $\operatorname{IVI}(C,f)$, where *C* is given as in (22).

### Algorithm 5.1

(The alternating contraction relaxation projection method)

1. Take $u_{0}\in C$ and $\xi _{0}^{(0)}\in \mathcal{H}$ arbitrarily and set $n:=0$.
2. For the current $u_{n}$ and $\xi _{n}^{(0)}$ ($n\geq 0$), calculate
  23 $$ \xi ^{(m+1)}_{n}=\xi ^{(m)}_{n}- \alpha f\bigl(\xi ^{(m)}_{n} \bigr)+\alpha u_{n}, \quad m=0,1,\ldots,m _{n}, $$
  where $m_{n}$ is the smallest positive integer such that
  24 $$ \frac{(1-\tau )^{m_{n}+1}}{\tau } \bigl\Vert \xi ^{(1)}_{n}- \xi ^{(0)}_{n} \bigr\Vert \leq \varepsilon _{n}, $$
  where $\tau =\frac{1}{2}\alpha (2\eta -\alpha L^{2})$.
  Set
  25 $$ \xi _{n}=\xi ^{(m_{n}+1)}_{n}. $$
3. Calculate
  26 $$ u_{n+1}=P_{C_{n}}(u_{n}-\lambda _{n}\mu \xi _{n}), $$
  where
  27 $$\begin{aligned}& \begin{aligned}[b] &C_{n}=\bigl\{ z\in \mathcal{H}\mid c(u_{n})+ \langle \nu _{n}, z-u_{n}\rangle \leq 0\bigr\} , \\&\quad \nu _{n}\in \partial c(u_{n}) \quad \text{and} \quad \lambda _{n}\in (0, 1).\end{aligned} \end{aligned}$$
  Set
  $$ \xi ^{(0)}_{n+1}=\xi _{n}, $$
  $n:=n+1$ and return to Step 2.

Next theorem establishes the strong convergence of Algorithm 4.1.

### Theorem 5.1

*Let* *C* *be given by* (22), *and let* $f: \mathcal{H}\rightarrow \mathcal{H}$ *be an* *L*-*Lipschitz continuous and* *η*-*strongly monotone operator*. *Assume that* $c: \mathcal{H}\rightarrow \mathbb{R}$ *is weakly lower semi*-*continuous and subdifferentiable on* $\mathcal{H}$ *and* *∂c* *is a bounded operator*. *Suppose that the sequence* $\{\lambda _{n}\}_{n=0}^{\infty }\subset (0, 1)$ *satisfies* (i) $\lambda _{n}\rightarrow 0$ *as* $n\rightarrow \infty $ *and* (ii) $\sum_{n=0}^{\infty }\lambda _{n}=\infty $. *Then the two sequences* $\{\xi _{n}\}_{n=0}^{\infty }$ *and* $\{u_{n}\}_{n=0}^{ \infty }$ *generated by Algorithm *5.1 *converge strongly to the unique solution* $\xi ^{*} $ *of* $\operatorname{IVI}(C,f)$ *and the unique solution* $u^{*}$ *of* $\operatorname{VI}(C,f^{-1})$, *respectively*.

### Proof

For convenience, we denote by *M* a positive constant, which represents different values in different places. Firstly, we verify that $\{u_{n}\}_{n=0}^{\infty }$ is bounded. Indeed, from the subdifferential inequality (7) and the definition of $C_{n}$, it is easy to verify that $C_{n}\supset C$ for all $n\geq 0$. Similar to (15), we also have

28 $$ \bigl\Vert \xi _{n}-f^{-1}(u_{n}) \bigr\Vert \leq \varepsilon _{n}, \quad n\geq 0. $$

Noting that the projection operator $P_{C_{n}}$ is nonexpansive, we obtain from (26), (28), and Lemma 2.4 that

$$ \begin{aligned} \bigl\Vert u_{n+1}-u^{*} \bigr\Vert &= \bigl\Vert P_{C_{n}}(u_{n}-\lambda _{n} \mu \xi _{n})-P _{C_{n}}u^{*} \bigr\Vert \\ &= \bigl\Vert P_{C_{n}}(u_{n}-\lambda _{n}\mu \xi _{n})-P_{C _{n}}\bigl(u_{n}-\lambda _{n} \mu f^{-1}(u_{n})\bigr) \bigr\Vert \\ &\quad {} + \bigl\Vert P_{C_{n}}\bigl(u_{n}- \lambda _{n} \mu f^{-1}(u_{n})\bigr)-P_{C_{n}}u^{*} \bigr\Vert \\ &\leq \bigl\Vert P_{C_{n}}\bigl(u _{n}-\lambda _{n}\mu f^{-1}(u_{n})\bigr)-P_{C_{n}} \bigl(u^{*}-\lambda _{n}\mu f ^{-1} \bigl(u^{*}\bigr)\bigr) \bigr\Vert \\ & \quad {} + \bigl\Vert P_{C_{n}}\bigl(u^{*}-\lambda _{n}\mu f^{-1}\bigl(u^{*} \bigr)\bigr)-P_{C_{n}}u^{*} \bigr\Vert +\lambda _{n}\mu \varepsilon _{n} \\ &\leq (1- \tilde{\tau }\lambda _{n}) \bigl\Vert u_{n}-u^{*} \bigr\Vert +\tilde{\tau }\lambda _{n}\frac{ \mu }{\tilde{\tau }}\bigl(\varepsilon _{n}+ \bigl\Vert f^{-1}\bigl(u^{*}\bigr) \bigr\Vert \bigr) \\ &\leq (1- \tilde{\tau }\lambda _{n}) \bigl\Vert u_{n}-u^{*} \bigr\Vert +\tilde{\tau }\lambda _{n}\frac{ \mu }{\tilde{\tau }}M, \end{aligned} $$

where $\tilde{\tau }=\frac{1}{2}\mu (2\tilde{\eta }-\mu \tilde{L}^{2})$. Inductively, it turns out that

$$ \Vert u_{n}-u \Vert \leq \max \biggl\{ \Vert u_{0}-u \Vert ,\frac{\mu }{\tilde{\tau }} M\biggr\} , \quad \forall n\geq 1, $$

which implies that $\{u_{n}\}_{n=0}^{\infty }$ is bounded and so is $\{f^{-1}(u_{n})\}_{n=0}^{\infty }$. Similar to (17), we have

29 $$ \begin{aligned}[b] \bigl\Vert \xi _{n}- \xi ^{*} \bigr\Vert &\leq \bigl\Vert \xi _{n}-f^{-1}(u_{n}) \bigr\Vert + \bigl\Vert f^{-1}(u_{n})-\xi ^{*} \bigr\Vert \\ &\leq \varepsilon _{n}+ \bigl\Vert f^{-1}(u_{n})-f^{-1} \bigl(u^{*}\bigr) \bigr\Vert \\ & \leq \varepsilon _{n}+\tilde{L} \bigl\Vert u_{n}-u^{*} \bigr\Vert , \end{aligned} $$

which implies that $\{\xi _{n}\}_{n=0}^{\infty }$ is bounded. Using (26), (28), Lemma 2.1, and Lemma 2.4, we have

30 $$\begin{aligned} & \bigl\Vert u_{n+1}-u^{*} \bigr\Vert ^{2} \\ &\quad = \bigl\Vert P_{C_{n}}(u_{n}-\lambda _{n}\mu \xi _{n})-P _{C_{n}}u^{*} \bigr\Vert ^{2} \\ &\quad\leq \bigl\Vert u_{n}-\lambda _{n}\mu \xi _{n}-u^{*} \bigr\Vert ^{2} \\ &\quad= \bigl\Vert (u_{n}-\lambda _{n}\mu \xi _{n})-\bigl(u_{n}-\lambda _{n}\mu f^{-1}(u _{n})\bigr)+\bigl(u_{n}-\lambda _{n}\mu f^{-1}(u_{n})\bigr)-u^{*} \bigr\Vert ^{2} \\ &\quad\leq \bigl\Vert \bigl(u _{n}-\lambda _{n}\mu f^{-1}(u_{n})\bigr)-u^{*} \bigr\Vert ^{2}+2\lambda _{n}\mu \varepsilon _{n} \bigl\Vert u_{n}-\lambda _{n}\mu f^{-1}(u_{n})-u^{*} \bigr\Vert \\ &\quad\quad {} +\lambda ^{2}_{n}\mu ^{2}\varepsilon ^{2}_{n} \\ &\quad\leq \bigl\Vert \bigl(u_{n}-\lambda _{n}\mu f ^{-1}(u_{n})\bigr)-\bigl(u^{*}-\lambda _{n}\mu f^{-1}\bigl(u^{*}\bigr)\bigr)-\lambda _{n}\mu f ^{-1}\bigl(u^{*}\bigr) \bigr\Vert ^{2}+\lambda _{n}\varepsilon _{n} M \\ &\quad\leq (1- \tilde{\tau }\lambda _{n}) \bigl\Vert u_{n}-u^{*} \bigr\Vert ^{2}-2\lambda _{n}\mu \bigl\langle f^{-1}\bigl(u^{*} \bigr),u_{n}-u^{*}-\lambda _{n}\mu f^{-1}(u_{n})\bigr\rangle \\ &\quad\quad {} + \lambda _{n}\varepsilon _{n} M \\ &\quad=(1-\tilde{\tau }\lambda _{n}) \bigl\Vert u_{n}-u ^{*} \bigr\Vert ^{2}+\tilde{\tau }\lambda _{n} \biggl[\frac{1}{\tilde{\tau }}\bigl(-2\mu \bigl\langle f^{-1} \bigl(u^{*}\bigr),u_{n} -u^{*}\bigr\rangle \\ &\quad\quad {} +2\lambda _{n}\mu ^{2} \bigl\Vert f ^{-1} \bigl(u^{*}\bigr) \bigr\Vert \bigl\Vert f^{-1}(u_{n}) \bigr\Vert +\varepsilon _{n}M\bigr)\biggr]. \end{aligned}$$

Since the projection operator $P_{C_{n}}$ is firmly nonexpansive, we get

31 $$ \bigl\Vert P_{C_{n}}u_{n}-P_{C_{n}}u^{*} \bigr\Vert ^{2}\leq \bigl\Vert u_{n}-u^{*} \bigr\Vert ^{2}- \Vert u_{n}-P _{C_{n}}u_{n} \Vert ^{2}. $$

Using (28) and (31), we have

32 $$ \begin{aligned}[b] & \bigl\Vert u_{n+1}-u^{*} \bigr\Vert ^{2} \\ &\quad = \bigl\Vert P_{C_{n}}(u_{n}-\lambda _{n}\mu \xi _{n})-P _{C_{n}}u^{*} \bigr\Vert ^{2} \\ &\quad= \bigl\Vert P_{C_{n}}(u_{n}-\lambda _{n}\mu \xi _{n})-P _{C_{n}}\bigl(u_{n}-\lambda _{n} \mu f^{-1}(u_{n})\bigr) \\ &\quad\quad {} +P_{C_{n}}\bigl(u_{n}- \lambda _{n}\mu f^{-1}(u_{n})\bigr)-P_{C_{n}}u^{*} \bigr\Vert ^{2} \\ &\quad\leq \bigl\Vert P_{C_{n}}\bigl(u _{n}-\lambda _{n}\mu f^{-1}(u_{n})\bigr)-P_{C_{n}}u^{*} \bigr\Vert ^{2}+2\lambda _{n} \mu \varepsilon _{n} \bigl\Vert u_{n}-u^{*}-\lambda _{n}\mu f^{-1}(u_{n}) \bigr\Vert \\ &\quad\quad {} + \lambda ^{2}_{n}\mu ^{2}\varepsilon ^{2}_{n} \\ &\quad\leq \bigl\Vert P_{C_{n}}\bigl(u_{n}- \lambda _{n}\mu f^{-1}(u_{n})\bigr)-P_{C_{n}}u_{n}+P_{C_{n}}u_{n}-P_{C_{n}}u ^{*} \bigr\Vert ^{2}+\lambda _{n} \varepsilon _{n}M \\ &\quad\leq \bigl\Vert P_{C_{n}}u _{n}-P_{C_{n}}u^{*} \bigr\Vert ^{2}+2\lambda _{n}\mu \bigl\Vert f^{-1}(u_{n}) \bigr\Vert \bigl\Vert u_{n}-u ^{*} \bigr\Vert + \lambda ^{2}_{n}\mu ^{2} \bigl\Vert f^{-1}(u_{n}) \bigr\Vert ^{2} \\ & \quad\quad {} +\lambda _{n} \varepsilon _{n}M \\ &\quad\leq \bigl\Vert P_{C_{n}}u_{n}-P_{C_{n}}u^{*} \bigr\Vert ^{2}+\lambda _{n}M \\ &\quad\leq \bigl\Vert u_{n}-u^{*} \bigr\Vert ^{2}- \Vert u_{n}-P_{C_{n}}u_{n} \Vert ^{2}+\lambda _{n}M. \end{aligned} $$

Setting

$$\begin{aligned}& s_{n}= \bigl\Vert u_{n}-u^{*} \bigr\Vert ^{2}, \quad\quad \gamma _{n}=\tilde{\tau } \lambda _{n}, \\& \delta _{n}=-\frac{1}{\tilde{\tau }}\bigl(2\mu \bigl\langle f^{-1}\bigl(u^{*}\bigr),u_{n}-u ^{*}\bigr\rangle \bigr)+\frac{1}{\tilde{\tau }}\bigl(2\lambda _{n}\mu ^{2} \bigl\Vert f^{-1}\bigl(u ^{*}\bigr) \bigr\Vert \bigl\Vert f^{-1}(u_{n}) \bigr\Vert + \varepsilon _{n}M \bigr), \\& \eta _{n}= \Vert u_{n}-P_{C_{n}}u_{n} \Vert ^{2}, \quad\quad \alpha _{n}= M\lambda _{n}, \end{aligned}$$

then (30) and (32) can be rewritten as the following forms, respectively:

33 $$ s_{n+1}\leq (1-\gamma _{n})s_{n}+\gamma _{n}\delta _{n}, \quad n\geq 0, $$

and

34 $$ s_{n+1}\leq s_{n}-\eta _{n}+\alpha _{n}, \quad n\geq 0. $$

From the conditions $\lambda _{n}\rightarrow 0$ and $\sum^{+\infty }_{n=1} \lambda _{n}=\infty $, it follows $\alpha _{n} \rightarrow 0$ and $\sum^{\infty }_{n=1} \gamma _{n}=\infty $. So, in order to use Lemma 2.6 to complete the proof, it suffices to verify that

$$ \lim_{k\rightarrow \infty }\eta _{n_{k}}=0 $$

implies

$$ \limsup_{k\rightarrow \infty }\delta _{n_{k}}\leq 0 $$

for any subsequence $\{n_{k}\}_{k=0}^{\infty }\subset \{n\}_{n=0}^{ \infty }$. In fact, from $\Vert u_{n_{k}}-P_{C_{n_{k}}}u _{n_{k}} \Vert \rightarrow 0$ and the fact that *∂c* is bounded on bounded sets, it follows that there exists a constant $\delta > 0 $ such that $\Vert \nu _{n_{k}} \Vert \leq \delta $ for all $k\geq 0$. Using (27) and the trivial fact that $P_{C_{n_{k}}}u_{n_{k}} \in C_{n_{k}}$, we have

35 $$ c(u_{n_{k}})\leq \langle \nu _{n_{k}},u_{n_{k}}-P_{C_{n_{k}}}u_{n_{k}} \rangle \leq \delta \Vert u_{n_{k}}-P_{C_{n_{k}}}u_{n_{k}} \Vert . $$

For any $u^{\prime }\in \omega _{\omega }(u_{n_{k}})$, without loss of generality, we assume that $u_{n_{k}} \rightharpoonup u^{\prime }$. Using *w-lsc* of *c* and (35), we have

$$ c\bigl(u^{\prime }\bigr)\leq \liminf_{k\rightarrow \infty }c(u_{n_{k}}) \leq 0, $$

which implies that $u^{\prime }\in C$. Hence $\omega _{\omega }(u_{n_{k}})\subset C$.

Noting that $u^{*}$ is the unique solution of $\operatorname{VI}(C,f^{-1})$, it turns out that

$$ \begin{aligned} \limsup_{k\rightarrow \infty }\biggl\{ - \frac{2\mu }{\tau }\bigl\langle f^{-1}\bigl(u ^{*} \bigr),u_{n_{k}}-u^{*}\bigr\rangle \biggr\} = {}&-\frac{2\mu }{\tau } \liminf_{k\rightarrow \infty }\bigl\langle f^{-1}\bigl(u^{*} \bigr),u_{n_{k}}-u^{*} \bigr\rangle \\ ={}&-\frac{2\mu }{\tau } \inf_{\omega \in \omega _{\omega }(u_{n_{k}})}\bigl\langle f^{-1} \bigl(u^{*}\bigr), \omega -u^{*}\bigr\rangle \leq 0. \end{aligned} $$

Since $\lambda _{n}\rightarrow 0$, $\varepsilon _{n}\rightarrow 0$, and $\{f^{-1}(u_{n})\}_{n=0}^{\infty }$ is bounded, it is easy to verify that $\limsup_{k\rightarrow 0} \delta _{n_{k}}\leq 0$. Therefore, by using Lemma 2.6 we get that $u_{n}\rightarrow u^{*}$ as $n\rightarrow \infty $. Consequently, this together with (29) leads to $\xi _{n}\rightarrow \xi ^{*}$ and the proof is completed. □

Next we estimate the convergence rate of Algorithm 5.1. Note that the conditions $\lambda _{n}\rightarrow 0$ and $\sum_{n=0}^{\infty }\lambda _{n}=\infty $ guarantee the strong convergence, but slow down the convergence rate. Since it is difficult to estimate the asymptotic convergence rate of Algorithm 5.1, we will focus on the convergence rate of Algorithm 5.1 in the non-asymptotic sense. Based on Lemma 1.1 and Theorem 5.1, estimating the convergence rate of Algorithm 5.1 for $\operatorname{IVI}(C,f)$ is equivalent to estimating the convergence rate of Algorithm 5.1 for $\operatorname{VI}(C,f^{-1})$, so we will analyze the convergence rate of Algorithm 5.1 for $\operatorname{VI}(C,f^{-1})$.

The analysis of the convergence rate is based on the fundamental equivalence: a point $u\in C$ is a solution of $\operatorname{VI}(C,f^{-1})$ if and only if $\langle f^{-1}(v),v-u\rangle \geq 0$ holds for all $v\in C\cap S(u,1)$, where $S(u,1)$ is the closed sphere with the center *u* and the radius one (see [4] and [10] for details).

A useful inequality for estimating the convergence rate of Algorithm 5.1 is given as follows.

### Lemma 5.1

*Let* $\{u_{n}\}_{n=1}^{\infty }$ *be the sequence generated by Algorithm *5.1. *Assume that the conditions in Theorem *5.1 *hold*. *Suppose* $\sum_{n=0}^{\infty }\lambda _{n}^{2}<\infty $ *and* $\sum_{n=0}^{\infty }\lambda _{n}\varepsilon _{n}<\infty $. *Then*, *for any integer* $n\geq 1$, *we have a sequence* $\{w_{n}\}_{n=1}^{ \infty }$ *which converges strongly to the unique solution* $u^{*}$ *of* $\operatorname{VI}(C,f^{-1})$ *and*

36 $$ \begin{aligned}[b] & \bigl\langle f^{-1}(v),w_{n}-v \bigr\rangle \\ &\quad \leq \frac{ \Vert u _{0}-v \Vert ^{2}+\mu ^{2}(\sigma _{1}+\sigma _{2})+2\mu (\sigma _{3}+\sigma _{4} \Vert v \Vert +\mu \sigma _{5})}{\varUpsilon _{n}}, \quad \forall v\in C, \end{aligned} $$

*where*

37 $$ \begin{gathered} \sigma _{1}= \sum_{k=0}^{\infty }\lambda _{k}^{2} \bigl\Vert f^{-1}(u_{k}) \bigr\Vert ^{2}, \quad \quad \sigma _{2}=\sum_{k=0}^{\infty } \lambda _{k}^{2}\varepsilon _{k}^{2}, \\ \sigma _{3}=\sum_{k=0}^{\infty } \lambda _{k}\varepsilon _{k} \Vert u_{k} \Vert , \quad\quad \sigma _{4}=\sum_{k=0}^{\infty } \lambda _{k}\varepsilon _{k}, \\ \sigma _{5}=\sum_{k=0}^{\infty } \lambda _{k}^{2}\varepsilon _{k} \bigl\Vert f^{-1}(u_{k}) \bigr\Vert , \quad\quad w_{n}= \frac{\sum_{k=0}^{n}2\mu \lambda _{k}u_{k}}{\varUpsilon _{n}},\quad \textit{and} \quad \varUpsilon _{n}=\sum _{k=0}^{n}2\mu \lambda _{k}. \end{gathered} $$

### Proof

For each $k\geq 0$ and any $v\in C$, using (26) and (28), we have

$$ \begin{aligned}[b] \Vert u_{k+1}-v \Vert & = \bigl\Vert P_{C_{k}}(u_{k}-\lambda _{k}\mu \xi _{k})-P _{C_{k}}v \bigr\Vert \\ &\leq \Vert u_{k}-\lambda _{k}\mu \xi _{k}-v \Vert \\ &\leq \bigl\Vert u _{k}-v-\lambda _{k}\mu f^{-1}(u_{k})+\lambda _{k}\mu f^{-1}(u_{k})- \lambda _{k}\mu \xi _{k} \bigr\Vert \\ &\leq \bigl\Vert u_{k}-v-\lambda _{k}\mu f^{-1}(u _{k}) \bigr\Vert +\lambda _{k}\mu \varepsilon _{k}. \end{aligned} $$

Consequently, we obtain

38 $$ \begin{aligned}[b] & \Vert u_{k+1}-v \Vert ^{2} \\ &\quad \leq \Vert u_{k}-v \Vert ^{2}-2\lambda _{k}\mu \bigl\langle f^{-1}(u_{k}), u_{k}-v\bigr\rangle +\mu ^{2}\lambda _{k}^{2} \bigl\Vert f^{-1}(u_{k}) \bigr\Vert ^{2}+\mu ^{2}\lambda _{k}^{2}\varepsilon _{k}^{2} \\ &\quad\quad{} +2 \mu \lambda _{k}\varepsilon _{k} \Vert u_{k} \Vert +2\mu \lambda _{k}\varepsilon _{k} \Vert v \Vert +2\mu ^{2} \lambda _{k}^{2} \varepsilon _{k} \bigl\Vert f^{-1}(u_{k}) \bigr\Vert , \end{aligned} $$

which together with the monotonicity of $f^{-1}$ yields

39 $$ \begin{aligned}[b] &2\lambda _{k}\mu \bigl\langle f^{-1}(v), u_{k}-v\bigr\rangle \\ &\quad \leq \Vert u _{k}-v \Vert ^{2}- \Vert u_{k+1}-v \Vert ^{2} +\mu ^{2}\lambda _{k} ^{2} \bigl\Vert f^{-1}(u_{k}) \bigr\Vert ^{2}+\mu ^{2}\lambda _{k}^{2} \varepsilon _{k}^{2} \\ &\quad\quad {} +2\mu \lambda _{k}\varepsilon _{k} \Vert u_{k} \Vert +2\mu \lambda _{k}\varepsilon _{k} \Vert v \Vert +2\mu ^{2}\lambda _{k}^{2} \varepsilon _{k} \bigl\Vert f^{-1}(u_{k}) \bigr\Vert . \end{aligned} $$

Note the fact that $\{u_{k}\}_{k=0}^{\infty }$ and $\{ \Vert f^{-1}(u_{k}) \Vert \}_{k=0}^{\infty }$ are all bounded. So, from the conditions $\sum_{k=0}^{\infty }\lambda _{k}^{2}<\infty $ and $\sum_{k=0}^{\infty }\lambda _{k}\varepsilon _{k}<\infty $, it follows that $\sigma _{k}<\infty $, $k=1,2,3,4$. Summing inequality (39) over $k=0,\ldots,n$, we get

40 $$ \begin{aligned}[b] & \Biggl\langle f^{-1}(v), \sum_{k=0}^{n}2\mu \lambda _{k} u_{k}- \sum_{k=0}^{n}2\mu \lambda _{k} v \Biggr\rangle \\ &\quad \leq \Vert u_{0}-v \Vert ^{2}+\mu ^{2}( \sigma _{1}+\sigma _{2})+2\mu \bigl(\sigma _{3}+ \sigma _{4} \Vert v \Vert +\mu \sigma _{5}\bigr) \quad \forall v\in C. \end{aligned} $$

Thus (36) follows from (37) and (40).

By Theorem 5.1, $\{u_{n}\}_{n=0}^{\infty }$ converges strongly to the unique solution $u^{*}$ of $\operatorname{VI}(C,f^{-1})$. Since $w_{n}$ is a convex combination of $u_{0}, u_{1},\ldots, u_{n}$, it is easy to see that $\{w_{n}\}_{n=1}^{\infty }$ also converges strongly to $u^{*}$. □

Finally we are in a position to estimate the convergence rate of Algorithm 5.1.

### Theorem 5.2

*Assume that the conditions in Theorem *5.1 *hold and the condition* $\sum_{n=0}^{\infty }\lambda _{n}\varepsilon _{n}<\infty $ *is satisfied*. *Then*, *in the ergodic sense*, *Algorithm *5.1 *has the* $O (\frac{1}{n^{1-\alpha }} )$ *convergence rate if* $\{\lambda _{n}\}_{n=1}^{\infty }=\{\frac{1}{n^{\alpha }}\}_{n=1}^{ \infty }$ *with* $\frac{1}{2}<\alpha <1$ *and* $\lambda _{0}=\frac{1}{1- \alpha }$, *and has the* $O (\frac{1}{\ln n} )$ *convergence rate if* $\{\lambda _{n}\}_{n=1}^{\infty }=\{\frac{1}{n}\}_{n=1}^{ \infty }$.

### Proof

For $\{\lambda _{n}\}_{n=1}^{\infty }=\{\frac{1}{n^{ \alpha }}\}_{n=1}^{\infty }$ with $\frac{1}{2}<\alpha <1$, one has that $\sum_{n=1}^{\infty }\lambda _{n}=\infty $ and $\sum_{n=1}^{\infty } \lambda _{n}^{2}< \infty $. For any integer $k\geq 1$, it is easy to verify that

$$ \frac{1}{1-\alpha }\bigl\{ (k+1)^{1-\alpha }-k^{1-\alpha }\bigr\} \leq \frac{1}{k ^{\alpha }}. $$

Consequently, for all $n\geq 1$, we have

41 $$ \frac{1}{1-\alpha }\bigl\{ (n+1)^{1-\alpha }-1\bigr\} \leq \sum _{k=1}^{n}\frac{1}{k ^{\alpha }}. $$

It concludes from (37) and (41) that

42 $$ \varUpsilon _{n}\geq \frac{2\mu }{(1-\alpha )}(n+1)^{1-\alpha } \geq \frac{2 \mu }{(1-\alpha )}n^{1-\alpha }, $$

which implies that Algorithm 5.1 has the $O (\frac{1}{n^{1-\alpha }} )$ convergence rate. In fact, for any bounded subset $D\subset C$, put $\gamma =\sup \{ \Vert v \Vert \mid v\in D\}$, then from (36) and (42), we obtain

43 $$ \begin{aligned}[b] & \bigl\langle f^{-1}(v),w_{n}-v \bigr\rangle \\ &\quad \leq \frac{(1- \alpha )\{( \Vert u_{0} \Vert +\gamma ) ^{2}+\mu ^{2}(\sigma _{1}+\sigma _{2})+2 \mu (\sigma _{3}+\sigma _{4}\gamma +\mu \sigma _{5})\}}{2\mu n^{1-\alpha }}, \quad \forall v\in D. \end{aligned} $$

The conclusion can be similarly proved for $\{\lambda _{n}\}_{n=1}^{\infty }=\{\frac{1}{n}\}_{n=1}^{\infty }$. □

## Numerical experiments

In this section, in order to show the practicability and effectiveness of Algorithm 4.1 (ACPM), we present two examples in the setting of finite dimensional Hilbert spaces. The codes were written in Matlab 2009a and run on personal computer.

In the following two examples, we denote by $\{u_{n}\} _{n=0}^{\infty }$ and $\{\xi _{n}\}_{n=0}^{\infty }$ the two sequences generated by Algorithm 4.1. Take *L*, *η*, *α*, *μ*, *τ*, and *τ̃* as in Sect. 4 and $\varepsilon _{n}:=(1- \tilde{\tau })^{n+1}$ ($n\geq 0$). Since we do not know the exact solution $\xi ^{*}$ of $\operatorname{IVI}(C,f)$, we use $E_{n}=\frac{ \Vert \xi _{n+1}-\xi _{n} \Vert }{ \Vert \xi _{n} \Vert }$ to measure the error of the *n*th step iteration.

It is worth noting that for the following two examples, condition (6) is not satisfied, so the method proposed by Luo et al. [34] could not be used. However, Algorithm 4.1 can be implemented easily.

### Example 6.1

Let $f: \mathbb{R}^{1}\rightarrow \mathbb{R}^{1}$ be defined by

$$ f(x)=2x+\sin x, \quad \forall x\in \mathbb{R}^{1}, $$

and let $C=[1,10]\subset \mathbb{R}^{1}$. Obviously, *f* is 3-Lipschitz continuous and 1-strongly monotone. Hence, $L=3$ and $\eta =1$. Choose $\alpha =\mu =\frac{1}{9}$, $u_{0}=5$, $\xi _{0}^{(0)}=100$.

**Figure 1:**
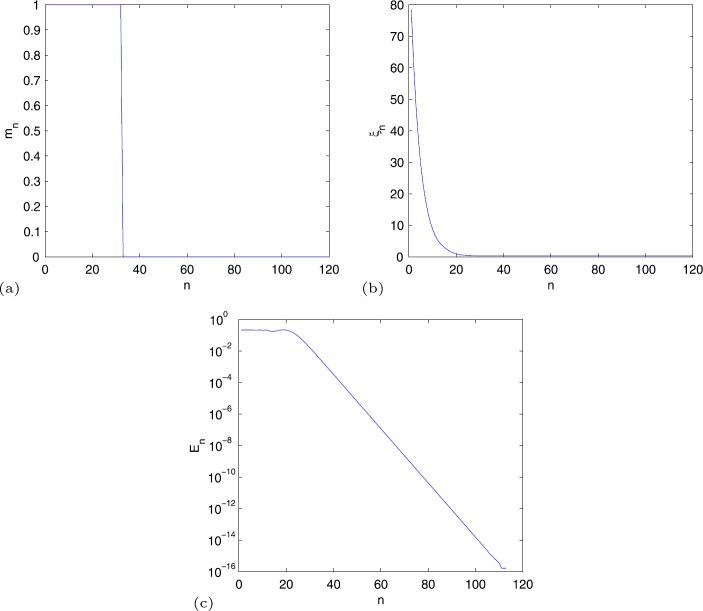
(**a**) $m_{n} $, (**b**) $\xi _{n}$, (**c**) $E_{n}$

The numerical results generated by implementing Algorithm 4.1 are provided in Fig. 1, from which we observe that

- $m_{n}$ is 1 when $n<33$ and becomes 0 when $n\geq 33$. Hence the calculation to find suitable $m_{n}$ is not needed when $n\geq 33$. This is a feature of our algorithms, which is different with other line search techniques.
- $\xi _{n}$ deceases with *n* and equals 3.3541803 when $n\geq 69$.
- Except the first steps, the error $E_{n}$ decreases linearly.

### Example 6.2

Let $f: \mathbb{R}^{2}\rightarrow \mathbb{R}^{2}$ be defined by

$$ f(x,y)=(2x+2y+\sin x, -2x+2y+\sin y)^{\top }, \quad \forall (x,y)^{\top }\in \mathbb{R}^{2}, $$

and let $C=[1,10]\times [1,10]\subset \mathbb{R}^{2}$. It is easy to directly verify that *f* is $3\sqrt{2}$-Lipschitz continuous and 1-strongly monotone. So we have $L=3\sqrt{2}$ and $\eta =1$. Select $\alpha =\mu =\frac{1}{18}$, $u_{0}=(3,4)^{\top }$, and $\xi _{0}^{(0)}=(20,20)^{ \top }$.

**Figure 2:**
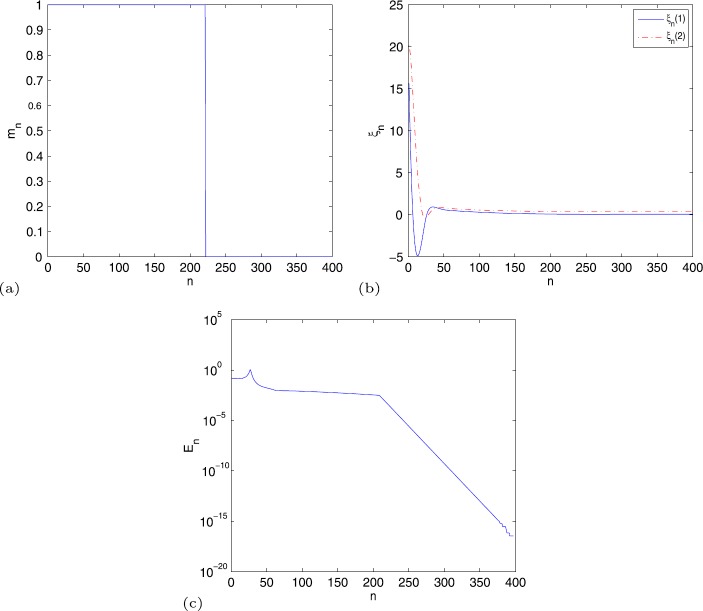
(**a**) $m_{n} $, (**b**) $\xi _{n}$, (**c**) $E_{n}$

From Fig. 2, we observe that: (a) $m_{n}$ is 1 when $n<222$ and $m_{n}$ becomes 0 when $n\geq 222$; (b) the vectors of $\xi _{n}$ do not decease as Example 6.1, and $\xi _{n}$ equals $[0.07546639, 0.38683623]^{T}$ when $n\geq 290$; (c) except the first steps, the decrease of error $E_{n}$ is piecewise linear.

## Concluding remarks

In this paper, we give an existence-uniqueness theorem for the inverse variational inequalities, whose conditions are weaker than those of Luo et al. [34]. Based on the existence-uniqueness theorem, we introduce an alternating contraction projection method and its relaxed version and show their strong convergence. The convergence rates of the alternating contraction projection method and its relaxed version are both presented. Comparing with the alternating contraction projection method, the convergence conditions of the alternating contraction relaxation projection method are stronger, but the alternating contraction relaxation projection method is indeed easy to implement when the projection operator $P_{C}$ is difficult to calculate.
